# Do Online Voting Patterns Reflect Evolved Features of Human Cognition? An Exploratory Empirical Investigation

**DOI:** 10.1371/journal.pone.0129703

**Published:** 2015-06-11

**Authors:** Maria Priestley, Alex Mesoudi

**Affiliations:** Department of Anthropology, Durham University, Durham, United Kingdom; University Toulouse 1 Capitole, FRANCE

## Abstract

Online votes or ratings can assist internet users in evaluating the credibility and appeal of the information which they encounter. For example, aggregator websites such as Reddit allow users to up-vote submitted content to make it more prominent, and down-vote content to make it less prominent. Here we argue that decisions over what to up- or down-vote may be guided by evolved features of human cognition. We predict that internet users should be more likely to up-vote content that others have also up-voted (social influence), content that has been submitted by particularly liked or respected users (model-based bias), content that constitutes evolutionarily salient or relevant information (content bias), and content that follows group norms and, in particular, prosocial norms. 489 respondents from the online social voting community Reddit rated the extent to which they felt different traits influenced their voting. Statistical analyses confirmed that norm-following and prosociality, as well as various content biases such as emotional content and originality, were rated as important motivators of voting. Social influence had a smaller effect than expected, while attitudes towards the submitter had little effect. This exploratory empirical investigation suggests that online voting communities can provide an important test-bed for evolutionary theories of human social information use, and that evolved features of human cognition may guide online behaviour just as it guides behaviour in the offline world.

## Introduction

The internet is becoming increasingly central to people’s lives. For many people who live in industrialised countries it is now a major means of acquiring and transmitting information, of social interaction and communication, of entertainment, and of buying and selling goods. With this increased use has inevitably come a proliferation of information. Internet users are faced with a barrage of news stories, articles, products, services and other content that they could never directly and exhaustively evaluate for themselves.

Consequently, many internet users rely on aggregator websites, or aggregation mechanisms built into other websites, to make decisions about what to read, view, buy, visit or endorse [1,2]. Dedicated aggregator websites such as Reddit (www.reddit.com) allow users to submit comments and posts that typically contain links to news stories, articles, images and videos found on other websites. These submissions can be up-voted or down-voted by other users to influence the content’s position and subsequent visibility. Similarly, retail websites such as Amazon (www.amazon.com) ask customers to evaluate other customers’ product reviews, and preferentially display reviews that have been rated the most helpful. Websites such as Reddit and Amazon are therefore using bottom-up, user-driven aggregated evaluations to filter information that would otherwise be overwhelming. This is in contrast to, say, traditional news sources such as print newspapers, which rely on editors to select content to present to readers in a top-down fashion.

This raises the question of how users decide to up-vote or down-vote content on such websites. In this exploratory empirical study we surveyed 489 Reddit users (known on the site as ‘Redditors’) asking them about their reasons for up- or down-voting content. Reddit was chosen because it is one of the most popular and prominent aggregator sites. It is currently in the top 50 of the world’s most visited websites [3], and it has over 100 million unique visitors per month, of which just over 3 million are registered users who cast over 20 million votes [4].

We are particularly interested in applying the novel framework of cultural evolutionary theory to online voting decisions. The field of cultural evolution [5–11] concerns (i) the way in which human cognition has biologically evolved to acquire, process and transmit information in an evolutionarily adaptive manner, such as by preferentially learning from successful individuals or by copying others only when one’s personal information is unreliable; and (ii) how these ‘social learning strategies’ [12,13] affect long-term cultural dynamics, i.e. how they influence change and variation in attitudes, beliefs, knowledge and other forms of culturally-transmitted information. Given that online voting is fundamentally concerned with the evaluation of information that originates from other people, and with the decision to transmit that information to others (by making it more or less prominent), we think it is plausible that psychological mechanisms that have evolved to serve these functions in the offline world may also apply to the online world. Note that there is strong overlap between cultural evolution and social psychology [14], and many of the same findings and predictions can be found in each tradition.

We therefore make the following general predictions concerning Redditors’ self-reported motivations for voting on content:

### Prediction 1: Social influence

Evolutionary models have formally examined the adaptiveness of *social learning*, defined as copying the knowledge or behaviour of other individuals, relative to *asocial learning*, defined as personally evaluating behaviours or knowledge with no influence from others [15]. One prediction of these models is that social learning should be used when asocial learning is particularly costly or ambiguous [16,17]. This prediction has received empirical support in both cultural evolution [18,19] and social psychology [20] experiments. Note that while this is a generally adaptive strategy, it can in certain cases lead to maladaptive ‘informational cascades’ where individuals copy inappropriate information from others without directly evaluating its effectiveness [21].

Given that it can be difficult to determine from its content alone whether a submission should be up- or down-voted, and also given that social information concerning others people’s voting decisions is freely available, we predict that Redditors should report using previous voting decisions as a guide to their voting decisions. A recent large-scale randomised experiment on a news aggregation website similar to Reddit [22] supports this prediction, finding that artificially up-voting a comment significantly increased the likelihood that actual users would subsequently up-vote that comment. Another study used replicate cultural markets to show that song preferences are susceptible to social influence, given that different songs became popular in different markets [23,24]. Our survey provides a test of whether Redditors are aware of the effect of social influence or not.

### Prediction 2: Informational content

Evolutionary models predict that people should preferentially copy and transmit useful or relevant information [6,16,25,26]. Empirical studies have identified various criteria for ‘usefulness’ or ‘relevance’ such as that the information concerns social interactions [27,28], contains supernatural or non-intuitive concepts [29,30], or elicits emotional reactions of disgust [31,32]. For our purposes, we might predict that Redditors up-vote content that has characteristics such as wide anticipated appeal or uniqueness. One recent study conducted textual analysis of user reviews on a retail website [33], finding that the rated helpfulness of a review is predicted by the review’s length, detail and understandability (e.g. use of short rather than long words). Our study represents a test of whether Redditors are aware of these effects. We might also expect Redditors to up-vote content that they personally agree with, and down-vote content that they personally disagree with, given evidence that people typically transform information to fit pre-existing beliefs [14,34].

### Prediction 3: Model-based bias

The fitness benefits of social learning can be further improved through selective learning from evidently skilful, successful or prestigious individuals, as they may be especially likely to possess useful information [12,35]. This has again been supported by experimental studies where participants preferentially copy successful individuals in various tasks [18,19,36,37] as well as copy individuals who others have looked at or shown deference in some way [38,39]. Here, we might predict that Redditors will report preferentially up-voting content that is submitted by individuals who they like and/or respect, and down-voting content that is submitted by individuals who they dislike and/or do not respect.

### Prediction 4: Group norm enforcement

Norms are defined as “learned behavioral standards shared and enforced by a community” [40] p.218. Social psychologists have long demonstrated the powerful role that norms play in directing human behaviour and judgement [41,42], while recent developmental psychology studies show that from a very early age children internalise and follow norms for even arbitrary behaviours [43]. Chudek and Henrich [40] argue that culture-gene coevolution has resulted in this powerful norm-psychology given that there were likely fitness benefits to both coordinating group behaviour, and adopting majority behaviours that represent the accumulated wisdom of previous generations.

Voting on Reddit takes place within an online community that can by itself instil particular shared norms in its users. Redditors are encouraged to adhere to an informal set of values known as the “reddiquette”, in addition to various moderated rules that guide user behaviour inside particular subreddits (subreddits are lists of submitted comments and links on specific topics, such as ‘worldnews’, ‘politics’ or ‘movies’). Given the possibility that people have evolved to enforce the behavioural standards of their community [40], it seems reasonable to expect that Redditors may internalise and try to enforce their community values by up-voting or down-voting content on the basis of its compliance with Reddit norms. We therefore predict that compliance with Reddit norms will be an important influence on Redditors’ voting decisions.

### Prediction 5: Enforcement of prosocial norms

Although in theory any behaviour can be stabilised as a social norm [44], there has been much interest in prosocial (or altruistic) norms which entail a cost to the individual and a benefit to the group [40]. Prosocial norms have been detected using economic games in which people willingly punish others who under-contribute to public goods [45]. This altruistic punishment has been observed across all human societies that have been studied [46,47], and especially in the Western societies from which the majority of our respondents come. One explanation for the widespread existence of prosocial norms is cultural group selection [6,16,40], wherein throughout human history groups with stable prosocial norms out-competed and replaced groups without such prosocial norms (although other theories based on purely individual benefit have also been proposed: [48]).

The occurrence of anti-social behaviour on the internet is well known (e.g. ‘trolling’). As noted above, Reddit is an established and successful online community, so we might expect Redditors to possess norms designed to prevent anti-social posts from disrupting that community. Our final prediction is therefore that Redditors will up-vote content that exhibits prosocial sentiments (e.g. praise or helpfulness), and down-vote content that exhibits anti-social sentiments (e.g. personal abuse).

### Overview

To summarise, our aim in this study is to examine whether people report their online voting to be motivated by (i) the votes of others via *social influence*, (ii) the *informational content* of posts, (iii) the characteristics of the poster, via *model-based bias*, (iv) the enforcement of *group norms*, and (v) the enforcement of *prosociality*. All of these predictions derive from prior models and lab experiments that aim to identify the adaptive design features of human information use. Some predictions have already been tested in an online voting context (e.g. [22,33]), although not using self-report surveys. While self-report surveys have many weaknesses, and people may well not be aware of the actual reasons behind their voting decisions [49], it is instructive to know the explicit motivations of online voters, and whether these match with the results of online experiments [22,23] and corpus analyses [33].

We take a two-stage approach to our survey. To avoid guiding our respondents towards the predictions above, we first take advantage of a subreddit called “Theory of Reddit” where frequent voters post their motivations for up- and down-voting content. From these we assembled a list of 29 commonly-stated reasons, which contained the predicted reasons but also a range of others. We then surveyed 489 Redditors to see which of these 29 were most important, how they clustered together, and whether the characteristics of Redditors (e.g. their age, gender, or time on the site) predicted their evaluation of each.

## Materials and Methods

### Ethics statement

The study was approved by the Durham University Department of Anthropology Research Ethics Committee. All participants viewed an informed consent web page and agreed to it by ticking an electronic box before proceeding with the study.

### Participants

Respondents were recruited through notices posted in subreddits that are concerned with how Reddit communities work (www.reddit.com/r/TheoryOfReddit), anthropology (www.reddit.com/r/Anthropology) and surveys (www.reddit.com/r/SampleSize). These subreddits were chosen because their users were expected to be interested in being part of the study. Participants were invited to follow a link to an online survey, which was hosted between 12^th^ to 20^th^ November 2013 using SurveyGizmo (www.surveygizmo.com) survey software. The sample consisted of 489 Redditors (236 females, 248 males, 5 gendered as “other”). The participants had a mean age of 26 years (s.d. = 7.78) with an age range of 18 to 64. The majority of participants were located in the United States (68.1%), Canada (8.0%), United Kingdom (5.9%) or Australia (3.5%), with the remaining 14.5% coming from 33 countries none of which individually exceeded 1.6% (8 participants).

### Materials

We distributed a survey to each participant that contained qualitative and quantitative questions in order to gain an insight into Redditors' motivations for voting, as well as brief demographic questions (see S1 File for full survey text). The first qualitative part of the survey asked participants to vote on a sample of 15 Reddit comments and then to write brief explanations for these decisions. The quantitative part of the survey contained generic questions about what influences the respondents’ usual voting behaviour on Reddit. This paper focuses only on the quantitative results from the second part of the survey.

During the first phase, the search term “upvote downvote” was used inside the subreddit “Theory of Reddit” to look for posts where Redditors previously discussed their motivations for voting. Search results were sorted by relevance and four posts whose titles referred to motivations for voting were opened. One of the researchers (MP) then read and analysed these posts and their response comments, which included a total of 180 comments made by Reddit users. A dramaturgical qualitative coding framework described in [50] was used to consider the users’ objectives, conflicts, tactics, attitudes, emotions and subtexts when analysing their written motivations for voting. This preliminary investigation identified 29 recurring themes upon which the quantitative questions in the survey were based. These characteristics encompassed traits that were necessary to address the predictions (e.g. content sounding intelligent, agreement or disagreement, aggression or consideration for other people etc.), as well as other unanticipated qualities. Participants were asked to rate the importance of these 29 characteristics as influences on upvotes and downvotes, placing their answers on a 5 point Likert scale ranging from “not important” to “very important”.

In addition to questions about voting motivations, respondents were asked to state their age, gender and Reddit membership duration category (1–6 months, 7–12 months, 1–2 years, 2–5 years, more than 5 years). They were also asked to state their frequency of Reddit visits, votes on posts, votes on comments, submissions of posts and submissions of comments on a four-point scale (daily, weekly, monthly, few times a year or less). Two variables were then created to represent the mean frequency of post and comment votes, and the mean frequency of post and comment submissions. The respondents’ location country was provided by SurveyGizmo. Respondents were asked to state the extent to which their overall voting decisions are influenced by emotional reactions to content, objective evaluations of the content’s quality, online reputation of the poster, and the length of time the poster had been a Redditor, where answers were given on a 5 point Likert scale ranging from “not at all” to “a lot”. Finally, there were two yes/no questions that asked respondents if they take more notice of highly up-voted content and content that is accompanied by Gold badges, which can act as indicators of commendation or popularity on Reddit.

### Analyses

All analyses were conducted using R version 2.8.0 [51]. First, a correlation matrix was calculated for the 29 variables concerned with different content characteristics as influences on voting. Principal Components Analysis (PCA) was used to explore whether these responses were organised in ways that reflected a smaller number of broad underlying forces, using the ‘principal’ function in R package *psych* [52]. The number of extracted factors was based on Eigenvalues greater than 1, and orthogonal (varimax) rotation was applied to better identify each item with a single factor.

Factor scores were created for each individual by calculating the mean of the raw scores corresponding to all items loading on a factor, as recommended for exploratory studies such as this one [53]. Consequently, factor scores retained the scale metric of the original Likert items, allowing for easier interpretation in subsequent analyses. These factor scores were then used as dependent variables in a series of ordinal logistic regression models to see if the importance scores of different voting influences varied depending on the voters' personal traits. Ordinal logistic regression was used because the dependent variables were 5-point Likert responses and were therefore not normally distributed. We used the clm and clmm functions in the R package ordinal [54] (clm gave identical results to the more popular polr function for ordinal logistic regression, but also allowed us to model random effects via the clmm function). Thirteen predictors were included in the models. Continuous predictors were the frequency of the respondent’s Reddit (i) visits, (ii) votes and (iii) contributions, (iv) their age, and the extent to which respondents report their voting being influenced by (v) their emotional reaction to the post, (vi) their objective evaluation of the post, (vii) the poster’s reputation, and (viii) the membership duration of the poster. Categorical predictors were the respondents' (ix) location country, (x) gender, (xi) Reddit membership duration, and whether they take more notice of (xii) up-voted content and (xiii) gold-badged content. Country was entered as a random effect given that shared location may generate non-independence in responses. For gender, ‘male’ served as the baseline against which ‘female’ and ‘other’ were compared. For Reddit membership duration, ‘1–6 months’ served as the baseline against which ‘6–12 months’, ‘1–2 years’, ‘2–5 years’ and ‘more than 5 years’ were compared. To avoid inflated Type I error rates associated with stepwise regression [55], we ran an initial full model with all thirteen predictors and then removed predictors with p>0.05. Model comparison was then used to check that all remaining predictors significantly improved model fit; where they did not they were removed. The remaining best-fit models are presented here. The full data file is available as S1 Dataset.

## Results

**Fig 1:**
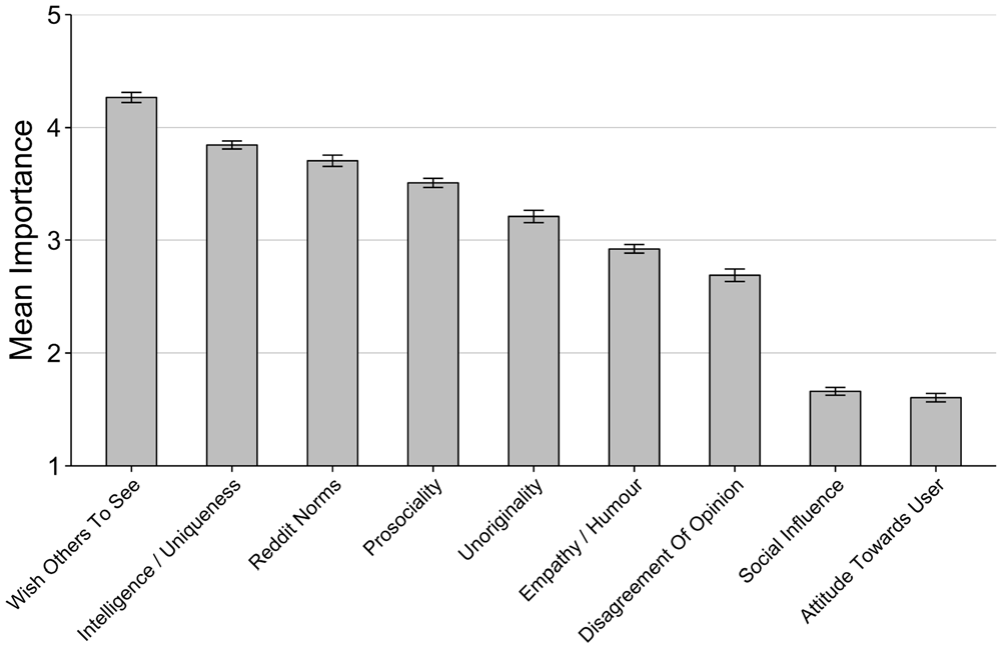
Mean importance of emergent influences on voting. Values are mean Likert responses on a scale of 1–5. Error bars indicate standard error.

The full list of 29 content characteristics selected for inclusion in the survey can be seen in the left-hand column of Table 1. These were all deemed appropriate for PCA analysis, since the Kaiser-Meyer-Olkin measure of sampling adequacy was 0.8 and Bartlett’s test of sphericity was highly significant (p<0.001). Eight principal components were extracted and they explained 61.85% of the total variance, as shown in Table 1. We labelled these factors *Reddit Norms* (whether content follows the rules of Reddit), *Empathy / Humour* (whether content elicits empathy, agreement or humour), *Social Influence* (the content’s existing number of up-votes or down-votes), *Prosociality* (whether content is considered socially damaging, rude or inconsiderate), *Intelligence / Uniqueness* (whether the content sounds intelligent, interesting or unique), *Unshared Experiences / Bad Memories*, *Unoriginality*, and *Attitude Towards User* (whether the content is posted by a liked or disliked user). Two variables remained unfactored, *Disagreement Of Opinion* and *Wish Others To See* (whether the voter thinks that the content should be viewed by other users). After the calculation of mean importance scores for each factor (see S1 Table), the factor *Unshared Experiences / Bad Memories* was dropped from further analyses due to low importance. Fig 1 shows the mean importance scores for the seven remaining factors and the two unfactored variables.

**Table 1:** Principal Components Analysis for 29 importance score items.

Principal component	1	2	3	4	5	6	7	8
Eigenvalue	3.28	2.85	2.55	2.54	1.82	1.69	1.67	1.55
Proportion of variance explained	0.11	0.10	0.09	0.09	0.06	0.06	0.06	0.05
*Reddit Norms*								
Follows subreddit rules (U)	.85							
Doesn't follow subreddit rules (D)	.83							
Irrelevant to post or subreddit (D)	.80							
Relevant to post or subreddit (U)	.76							
*Empathy / Humour*								
Shared experiences (U)		.75						
Humour (U)		.70						
Elicits sympathy or support (U)		.65						
Agreement of opinion (U)		.64						
Generally accepted opinion (U)		.54						
*Social Influence*								
Number of downvotes (U)			.76					
Number of upvotes (U)			.74					
Number of upvotes (D)			.73					
Number of downvotes (D)			.73					
*Prosociality*								
Immoral or socially damaging (D)				.82				
Rude or aggressive (D)				.69				
Consideration for others (U)				.54				
Bad humour (D)				.52				
Shouldn't be seen by others (D)				.52				
*Intelligence / Uniqueness*								
Sounds intelligent (U)					.79			
Sounds unintelligent (D)					.71			
Interesting/unique perspective (U)					.58			
*Unshared Experiences / Bad Memories*								
Unshared experience (D)						.79		
Brings back bad memories (D)						.77		
*Unoriginality*								
Expects upvotes (D)							.72	
Reposted or unoriginal (D)							.68	
*Attitude Towards User*								
Posted by a user the voter dislikes (D)								.73
Posted by a user the voter likes (U)								.65
*Wish Others To See* (U)								
*Disagreement Of Opinion* (D)								

Variables relate to upvotes (U) and downvotes (D). Factor loadings between +/-0.5 omitted. Proposed names for each emergent factor are italicised. *Disagreement Of Opinion* and *Wish Others To See* remained as unfactored variables.

We then regressed factor scores on our individual difference variables, to better understand which kind of Reddit user valued each factor. Table 2 shows significant predictors from the best-fitting regression model for each factor. We provide odds ratios (ORs) and their confidence intervals rather than beta coefficients as ORs are the most easily interpreted effect size measure from ordinal logistic regressions. ORs indicate the relative change in the odds of different outcomes occurring per unit change in a predictor. An OR = 1 indicates no change, and thus no effect. An OR = 1.10 for, say, the age predictor indicates that for every one-unit increase in age (by one year), the odds of choosing one level of the Likert-scale outcome variable (e.g. 5 = “Very important”) is 1.10 times the odds of choosing any lower level (e.g. 1 = “Not important”, 2, 3 or 4). Note that we cannot directly compare ORs for predictors in the same model that have different scales. For example, age is continuous ranging from 18–64, while gender has three categories, so a one-unit increase in age is different to a shift from male to female or other.

**Table 2:** Factor scores regressed on individual difference variables.

	OR	95% CI	z	*p*
*Reddit Norms*				
Age	0.97	[0.95, 0.99]	-2.47	0.014*
Gender (female)	1.51	[1.10, 2.08]	2.53	0.011*
Reddit membership (1–2 years)	2.09	[1.04, 4.17]	2.07	0.039*
Reddit membership (2–5 years)	2.62	[1.30, 5.26]	2.70	0.007**
Reddit membership (>5 years)	2.72	[1.03, 7.26]	2.01	0.044*
Objective evaluation	1.56	[1.31, 1.86]	5.04	<0.001***
Frequency of votes	1.57	[1.21, 2.05]	3.38	<0.001***
Frequency of submissions	1.41	[1.11, 1.78]	2.82	0.005**
*Empathy / Humour*				
Gender (female)	1.46	[1.06, 2.02]	2.32	0.021*
Emotional reaction	2.36	[2.01, 2.78]	10.43	<0.001***
Frequency of votes	1.42	[1.11, 1.81]	2.81	0.005**
*Prosociality*				
Emotional reaction	1.78	[1.53, 2.08]	7.33	<0.001***
Objective evaluation	1.23	[1.03, 1.46]	2.35	0.019*
Frequency of votes	1.57	[1.24, 1.98]	3.81	<0.001***
*Intelligence / Uniqueness*				
Gender (other)	0.15	[0.03, 0.72]	-2.40	0.016*
Frequency of contributions	1.38	[1.11, 1.71]	2.90	0.004**
Emotional reaction	1.30	[1.11, 1.52]	3.32	<0.001***
Objective evaluation	1.70	[1.43, 2.03]	5.92	<0.001***
*Unoriginality*				
Age	0.97	[0.95, 0.99]	-2.59	0.010**
Frequency of votes	1.61	[1.26, 2.07]	3.81	<0.001***
Reddit membership (1–2 years)	2.51	[1.25, 5.03]	2.60	0.009**
Reddit membership (2–5 years)	2.44	[1.22, 4.89]	2.52	0.012*
Reddit membership (>5 years)	3.20	[1.19, 8.61]	2.31	0.021*
Objective evaluation	1.44	[1.22, 1.71]	4.28	<0.001***
Notice gold badged content (No)	0.68	[0.49, 0.93]	-2.38	0.017*
Membership duration of poster	1.35	[1.07, 1.73]	2.49	0.013*
*Social Influence*				
Age	0.96	[0.94, 0.98]	-3.38	<0.001***
Emotional reaction	1.21	[1.03, 1.41]	2.36	0.018*
Frequency of votes	1.37	[1.02, 1.84]	2.09	0.037*
Frequency of submissions	1.50	[1.17, 1.93]	3.14	0.002**
Notice upvoted content (No)	0.44	[0.24, 0.76]	-2.84	0.004**
Reputation of poster	1.55	[1.24, 1.94]	3.84	<0.001***
*Wish Others To See*				
Objective evaluation	1.32	[1.10, 1.59]	2.94	0.003**
Emotional reaction	1.34	[1.13, 1.59]	3.41	<0.001***
Frequency of votes	1.63	[1.27, 2.11]	3.79	<0.001***
*Disagreement Of Opinion*				
Emotional reaction	1.68	[1.44, 1.96]	6.53	<0.001***
*Attitude Towards User*				
Emotional reaction	1.30	[1.10, 1.55]	2.98	0.003**
Frequency of submissions	1.58	[1.25, 2.02]	3.73	<0.001***
Reputation of poster	2.60	[2.07, 3.30]	8.03	<0.001***
Membership duration of poster	1.49	[1.14, 1.95]	2.91	0.004**

Significance codes:

p<0.001: ‘***’,

p<0.01: ‘**’,

p<0.05: ‘*’.

OR = Odds Ratios, CI = Confidence Intervals

The country variable was entered as a random effect in all models. However, none of these multi-level models showed significantly better fit compared to a model without country as a random effect. This indicates that country did not influence responses, most likely because the majority of our respondents were in Western countries (see Methods). The models in Table 2 therefore do not contain country as a random effect.

Table 2 shows that *Reddit Norms* was rated as more important by younger respondents, women, longer-term Reddit members, respondents who evaluated posts based on objective criteria, more frequent voters, and more frequent contributors. *Empathy / Humour* was rated higher by women, by respondents who report evaluating posts based on emotional reaction, and more frequent voters. *Prosociality* was rated as more important by more frequent voters, and by respondents who use both objective evaluations and emotional reactions. Emotional reaction (OR = 1.78, 95% CI [1.53, 2.08]) had a larger effect than objective evaluation (OR = 1.23, 95% CI [1.03, 1.46]), with non-overlapping confidence intervals (note that these predictors are comparable because they were measured on the same scales). *Intelligence / Uniqueness* was rated as more important by more frequent contributors and by respondents who use both objective evaluation and emotional reaction. Respondents reporting their gender as “other” appear to give lower importance compared to males, but this finding is unlikely to be reliable as the number of respondents who identified with this gender was very small (n = 5). Objective evaluation (OR = 1.70, 95% CI [1.43, 2.03]) had a larger effect than emotional reaction (OR = 1.30, 95% CI [1.11, 1.52]), although with slightly overlapping confidence intervals. *Unoriginality* was rated more important by younger respondents, longer-term Reddit members, more frequent voters, by respondents who use objective evaluation, by respondents who took notice of gold-badged content, and by respondents who use the membership duration of the poster. *Social Influence* was rated more important by younger respondents, respondents who use emotional reaction, more frequent voters and posters, respondents who notice upvoted content, and respondents who use the reputation of the poster. *Wish Others To See* was rated as more important by respondents who use objective evaluation and emotional reaction (with similar effect sizes), and more frequent voters. *Disagreement Of Opinion* was rated as more important only by respondents who use emotional reaction. Finally, *Attitude Towards User* was rated as more important by respondents who use emotional reaction, more frequent posters, and respondents who use the reputation and membership duration of poster. Of the latter, reputation (OR = 2.60, 95% CI [2.07, 3.30]) had a larger, non-overlapping effect size than membership duration (OR = 1.49, 95% CI [1.14, 1.95]).

Following the discovery of a significant relationship between age and *Social Influence*, additional tests were used to see if there were significant age differences in other questions associated with this variable. Mann-Whitney U tests showed that people who answered “yes” to “Do you take more notice of highly upvoted content?” were significantly younger (mean rank = 239.08) than those who answered “no” (mean rank = 288.11; z = -2.51, p < .05). Similarly, people who answered “yes” to “Do you take more notice of content accompanied by Gold badges?” were significantly younger (mean rank = 223.63) than those who answered “no” (mean rank = 266.64; z = -3.37, p < .05). For other possible markers of social influence, Spearman's Rank Order correlations showed that correlations between age and the perceived influence of the poster’s reputation (r_s_ = -.071, p > .05) and the poster’s membership duration (r_s_ = -.011, p > .05) were non-significant.

Descriptive analyses showed that content creators’ reputation and Reddit membership duration did not have a big perceived influence on the participants’ voting decisions, eliciting mean influence scores of 1.42 (s.d. = 0.78) and 1.25 (s.d. = 0.65) respectively on a 5 point Likert scale.

### Discussion

The aim of this study was to explore the self-reported motivations of frequent content voters on the popular aggregator website Reddit. We were particularly interested in whether these motivations might reflect features of human cognition and learning that have evolved in the offline world to process social information in an adaptive manner, as predicted by the formal models and experiments of cultural evolutionary theory.

An initial qualitative review of subreddit discussions in which Redditors themselves proposed reasons for up- and down-voting content yielded 29 potential motivations. These were then used in a quantitative survey of 489 active Redditors to assess their importance in a much larger sample, and identify individual differences between Redditors in their ratings of each. Principal Components Analysis revealed eight clusters of reasons. In descending order of perceived importance, these were *Wish Others To See*, *Intelligence / Uniqueness*, *Reddit Norms*, *Prosociality*, *Unoriginality*, *Empathy / Humour*, *Disagreement Of Opinion*, *Social Influence* and *Attitude Towards User*. The factor *Unshared Experiences / Bad Memories* was dropped due to low importance.

Some of these factors clearly map onto the predicted motivations for information sharing identified in the Introduction based on evolutionary principles. The factor *Social Influence* was predicted (Prediction 1) based both on theoretical analyses of the costs and benefits of social information use [15–17] and previous experiments showing social influence in online voting decisions [22]. However, *Social Influence* was here reported to be relatively unimportant compared to other factors (see Fig 1), with a mean importance score of just 1.66 (sd = 0.76) on a scale of 1–5. Previous randomized experiments [22] have shown that artificially introducing positive up-votes increased the chance of subsequent positive ratings by 32%, a not insubstantial effect. This mismatch may be because Redditors, like people in general [49], are unaware of the power of social influence over their behaviour. This combination of strong effect yet lack of awareness in those affected raises ethical questions over the power that social media websites, advertisers and other organisations may have over our online behaviour. We also found that younger respondents attributed a greater importance to *Social Influence*, a finding that was further supported by results showing that younger respondents were significantly more likely to report taking notice of highly up-voted content and Gold badges. It may be that younger people are simply more aware of the effect of social influence. Alternatively, younger people may genuinely be more susceptible to social influence, a possibility that deserves further study. As would be expected, and confirming the validity of our measures, respondents who attributed a greater importance to *Social Influence* also reported taking more notice of up-voted content and the reputation of the poster.

The following of group norms (Prediction 4) was reflected in the factor *Reddit Norms*, which concerned whether the submission obeyed the rules of, and is relevant to, the particular subreddit. Obviously human cognition has not biologically evolved to follow the rules of subreddits specifically, but evidence suggests that we *have* evolved to readily absorb and follow whatever local social norms we encounter [40–43]. Our findings reinforce this notion. *Reddit Norms* was rated of high importance (mean = 3.71, sd = 1.11; see Fig 1). Interestingly, norm compliance was rated as more important by younger respondents, longer-serving Redditors, and more frequent voters and posters. The effect of age is similar to that for *Social Influence*, with younger respondents more likely to acquire group norms via social learning. The other predictors may reflect an experience-dependent socialisation period, with more experienced Redditors having become more familiar with the norms of the Reddit community and thus more likely to have observed them in operation and being enforced. *Reddit Norms* was also rated as more important by female respondents, an unanticipated sex difference that deserves further investigation.

As predicted, norms specifically related to prosociality (Prediction 5) were rated as important by our respondents in the form of the *Prosociality* factor. Respondents were willing to up-vote (reward) submissions that reflect prosocial attitudes, and down-vote (punish) submissions that reflect abuse or aggression. Interestingly, the biggest individual difference variable that predicted *Prosociality* was whether the respondent used emotional responses as a guide to voting. This fits with recent studies showing that people are more cooperative when they use intuitive emotional processing rather than lengthy reflection and deliberation [45,56], and suggests that deep-seated evolved motivations for prosociality may shape online interactions.

Our second prediction (Prediction 2) concerned informational content, reflecting previous research suggesting that human cognition has evolved to bias information towards particularly salient representations, or ‘cultural attractors’, via ‘content biases’ [6,16,25,26,30]. In our study this appears to be reflected in more than one factor: *Wish Others To See*, *Intelligence / Uniqueness*, *Unoriginality*, *Empathy / Humour*, and *Disagreement Of Opinion* can all be seen as relating to the content of the submission. This suggests that there is no single criterion for informational content. The first factor, *Wish Others To See* (i.e. the up-voting of content that the respondent wants others to see), received the highest overall importance score (mean = 4.27, sd = 1.00), but is rather understandable given that this is the purpose of voting. This factor is not very theoretically informative given that it does not specify why the respondent wants content to be viewed by others, and how they select such content. *Intelligence / Uniqueness* and *Unoriginality* are more specific, suggesting that respondents use the perceived quality of a post as a guide to voting, e.g. whether it is intelligently coherent, or whether it is unique or original such that it will be novel to other Redditors. This reflects previous content analyses of up-voted reviews [33]. An important individual difference variable for both of these factors was whether the respondent uses objective criteria to guide their voting, which makes sense given that intelligence and originality can be objectively assessed.

*Empathy / Humour* also relates to the informational content of submissions, with respondents reporting being likely to up-vote content that refers to shared experiences, that elicits sympathy or support, that contains humour, or that the respondent agrees with (Table 1). Previous studies have shown that people are likely to remember and share emotionally salient information [31,32,57]. Individuals who report voting based on their emotional reaction to content give *Empathy / Humour* especially high importance. Female respondents also rated this factor as more important than male respondents, in line with previous behavioural and neurobiological findings that women show superior empathy than men [58] and women more than men find the production of humour attractive [59].

As part of Prediction 2 we expected that respondents should up-vote content that fits with their pre-existing attitudes and beliefs, and down-vote content they disagree with. In the study, however, agreement and disagreement did not cluster together: *Disagreement Of Opinion* factored on its own and was rated as relatively unimportant (mean = 2.69, sd = 1.23), while *Agreement* was grouped with *Empathy / Humour*. This may be a result of our self-report methodology, with respondents unwilling to admit to partisan voting based on their prior beliefs. However, it may be a genuine result driven by the community values that redditors are encouraged to adhere to, where down-voting based on personal opinion is particularly frowned upon.

Finally, our Prediction 3 that respondents would vote based on whether they like or respect the poster of the submission was not supported, since the factor *Attitude Towards User* was rated lowest in importance (mean = 1.60, sd = 0.83; see Fig 1). This is interesting given experimental evidence that people preferentially learn from high status, liked or knowledgeable individuals [35,38,39]. It may be that this is another influence that is outside explicit awareness. Alternatively, this may be a domain where content-based learning biases outweigh model-based learning biases. This is consistent with the finding that model traits such as the poster’s online reputation and Reddit membership duration were thought to be minimally influential by participants, whereas content-related traits were an important and recurring theme throughout the results.

It is important to acknowledge the limitations of our study. First, the reliance on self-report measures may not be representative of the true motivations behind voting behaviour, as we note above in relation to social influence. Second, the recruitment of respondents through targeted subreddits means that the sample may not be representative of the general user base of Reddit, or internet users in general. Third, the requirement to fill out a lengthy survey may have biased towards the recruitment of individuals with especially prosocial tendencies, which may have biased the results concerning prosociality in particular. For these reasons our findings should be treated with scepticism until they can be replicated with broader samples and more focused methods to address the emergent themes with greater precision.

In conclusion, this exploratory empirical study demonstrates that Redditors’ patterns of content voting can offer unique avenues of research into people’s interactions with information and its providers, and suggests a novel arena for testing evolutionary theories of social information use. Whereas expressions of fondness, agreement, aversions or punishments can be subjective in traditional settings, online votes provide clearly delineated forms of action that are well suited to empirical investigation.

## Supporting Information

S1 Dataset Survey data. The full data from the survey as a tab-delimited txt file.
(TXT)

S1 File Full survey. The full survey distributed to participants.
(PDF)

S1 Table Mean importance scores and standard deviations for voting influences. (DOC)
