# Supplementary material for: Do Online Voting Patterns Reflect Evolved Features of Human Cognition? An Exploratory Empirical Investigation
Source: PLoS One. 2015 Jun 11;10(6):e0129703. doi: 10.1371/journal.pone.0129703 (PMC4466230; doi:10.1371/journal.pone.0129703)
Supplement: S1 File — The full survey distributed to participants. (PDF) [file pone.0129703.s002.pdf]

Online rating behaviour

Page description:

You are about to participate in a study looking at online rating behaviour. This study is part of a student research project in the Department of Anthropology, Durham University.

To participate in this study you must be over the age of 18 and have used Reddit for at least one month.

In the first part of the questionnaire you will be asked to evaluate a number of Reddit comments. Some of the comments will contain swear words and potentially upsetting content. In the second part of the questionnaire you will be asked several questions about your personal voting behaviour.

The questionnaire should take approximately 20 minutes to complete and all of the information you provide will remain anonymous. You have the right to withdraw from the study at any time. If you choose to withdraw then your data will be removed from the study.

If you have any questions about this project feel free to contact maria.priestley@durham.ac.uk.

1. If you wish to be contacted with the results of this research once it is complete, please enter your email address below. (This is optional)

2. I have read all the information and agree to proceed with the study. *

☐ Confirm

New Page

3. Age (years) *

4. Gender *

5. Approximately how long have you been a member of Reddit? (including old or deleted accounts) *

- 1 to 6 months
- 7 to 12 months
- 1 to 2 years
- 2 to 5 years
- more than 5 years

6. How often do you *

	Daily	Weekly	Monthly	Few times a year or less
go on Reddit? *				
vote on Reddit posts? *				
vote on Reddit comments? *				
submit posts to Reddit? *				
make comments on Reddit submissions? *				

New Page

You will now be presented with a series of posts and response comments which have been sourced from Reddit. Please behave as you would on Reddit by rating or ignoring each of the response comments.

New Page

7. Please rate or ignore the response comment (shown in blue).

/r/AskReddit
Original poster:
Hey reddit, what kind of milk do you drink?
Responder:
I love milk, but I don't drink whole milk, I drink skim. *

- Upvote
- Downvote
- Ignore

Please give a brief explanation for your decision.

New Page

8. Please rate or ignore the response comment (shown in blue).

/r/Jokes
Original poster: Collection of my favorite Latvian Jokes.
Responder:
Two Latvian look at clouds.
One see potato. Other see impossible dream.
Is same cloud.
*

- Upvote
- Downvote
- Ignore

Please give a brief explanation for your decision.

New Page

9. Please rate or ignore the response comment (shown in blue).

/r/AskScienceDiscussion
Original poster: If You Put A Pineapple At The Bottom Of The Sea, What Kind Of Organisms, If Any, Would Actually Live In It?
Responder:
It depends how deep the bottom is. Because of the high pressure and cold temperatures, microbial action in the deep ocean is very slow. In 1968 the submersible Alvin accidentally sank in 1500 m of water when a cable snapped. No one was injured but the hatch was open and the crew's lunch for the day was packed inside the sphere. Ten months later, when the submersible was recovered, the lunch foods were in near perfect condition. Specific location wouldn't make as much difference as the depth (all the deep ocean is cold and high pressure) so I think the pineapple could last at least several years.
Sources:

- http://en.wikipedia.org/wiki/DSV_Alvin
- http://www.independent.co.uk/arts-entertainment/books/reviews/how-a-sandwich-unlocked-the-secrets-of-the-deep-625674.html

*

- Upvote
- Downvote
- Ignore

Please give a brief explanation for your decision.

New Page

10. Please rate or ignore the response comment (shown in blue).

/r/DebateReligion
Original poster: To Muslims: Is murder wrong?
Responder:
Murder is a terrible, terrible thing. It is only acceptable in defense of ones own life, or in defense of justice, and even then it is a terrible way to have somebody go.
And when I say justice I mean either to stop perpetual injustices from occurring i.e the murder of others, the forced slavery of others(not Islamic slavery but slavery like that of the earlier days of the US) and I mean as a punishment for murder. And even then only after non-violent means have been exhausted.
Also you should have no problem finding a Muslim saying something stupid, because Muslims are people as well, and finding a person say something stupid...well, look no further than this thread. In the Quran itself it says the majority of man do not reason, or something like that--it does not say the majority of non-muslims do not.
-Me, the Muslim.
*

- Upvote
- Downvote
- Ignore

Please give a brief explanation for your decision.

New Page

11. Please rate or ignore the response comment (shown in blue).

/r/funny
Original poster: Hello /r/funny! Let's have a groaner thread. Put your corny jokes here.
Responder:
Statistically, 6 out of 7 dwarfs are not Happy.
*

- Upvote
- Downvote
- Ignore

Please give a brief explanation for your decision.

New Page

12. Please rate or ignore the response comment (shown in blue).

/r/politics
Original poster:
"Education is the silver bullet... Schools should be palaces. Competition for the best teachers should be fierce; they should be making six figure salaries. Schools should be incredibly expensive for government and absolutely free of charge to its citizens." ~Rob Lowe, The West Wing
Responder:

Education is not the silver bullet. There may not be enough actual education going on in our society, but there are far too many educational degrees being printed, and far too many people paying far too much in tuition costs.

Ninety percent of jobs in this country are menial/stupid-- a psychological analogue of agricultural monoculture-- and it's worthless to educate people who will go on to those jobs; the only thing it will accomplish is making them really, really pissed-off, both due to the unrealistic promises made by their education (which prepare them for a career characterized by high creative control and autonomy) and the debt they took on to get that education.

We need sweeping changes in the whole society, not just the school system. We need to change our economy and the way people work. Unfortunately, there are a lot of very powerful people who have a vested interest in keeping society very, very stupid and we're probably not going to be able to improve things without going to war with them.

*

- Upvote
- Downvote
- Ignore

Please give a brief explanation for your decision.

New Page

13. Please rate or ignore the response comment (shown in blue).

/r/AskReddit
Original poster:
I was born into a Gypsy family... I have no money, no formal education, no work experience, and I am living in an environment that is increasingly hateful and abusive. I want to leave this house of many people and be on my own, but how?
Responder:
Don't ask for advice from reddit, it's full of the most smug self righteous assholes this side of the internet
*

- Upvote
- Downvote
- Ignore

Please give a brief explanation for your decision.

New Page

14. Please rate or ignore the response comment (shown in blue).

/r/AskReddit
Original poster:
So what's the worst thing that ever happened to you on your birthday?
Responder:
When I was about 8 years old, my cousin had a birthday party on my birthday at my house. I assumed it was a birthday party for both of us, and a lot of family and friends showed up.
The cake only had my cousin's name on it, but my family reassured me it was for both of us. Like five of the twenty-some people included my name in the Happy Birthday to You song; the rest just looked around confused as to why some people were messing up the song.
The only gift I got was from my mom, and I'm pretty sure she got it that day after remembering it was my birthday. I'll always be grateful for it.
My grandmother, always so cheerful, told me I should be grateful that I even got some of my cousin's cake.
Strangely enough though, it's one of the few birthdays I remember.
*

- Upvote
- Downvote
- Ignore

Please give a brief explanation for your decision.

New Page

15. Please rate or ignore the response comment (shown in blue).

/r/todayilearned
Original poster:
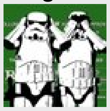
TIL advertisements used to be played after the movie - hence the name "trailer"
Responder:
Also the credits used to run before movies. Funny thing about the Star Wars thumbnail, it was one of the first movies to have the credits at the end.
*

- Upvote
- Downvote
- Ignore

Please give a brief explanation for your decision.

New Page

16. Please rate or ignore the response comment (shown in blue).

/r/Music
Original poster:
Modern rap music just repeats the same dumb shit over and over with a selfish greed filled message.
Responder:
This is so dumb. People seriously believe this shit? It's music not a fucking life or death kind of thing. Rap isn't going to kill you if you listen to it. If you don't like a certain genre of music don't listen to it. You're not cool because you don't like all modern music you just look stupid.

*

- Upvote
- Downvote
- Ignore

Please give a brief explanation for your decision.

New Page

17. Please rate or ignore the response comment (shown in blue).

/r/relationships
Original poster:
I need courage. How do I break up with my pregnant girlfriend?
tl;dr: I'm miserable in my relationship. My pregnant girlfriend and I fight over ridiculous things frequently. She acts completely childish over these fights and saying things like "I hate you, I'm breaking up with you, I'm kicking you out of the apartment". She has repeatedly stated how she will sue for child support and will take away my rights as a father. She has been in and out of a psyche ward for drug abuse. She destroyed some of my belongings in our latest fight. I feel I am being manipulated into staying but I don't have the courage to leave. I need advice.
Responder:
Is there some way you can go and get legal advice anywhere? I would say that is the first thing to do.
You do need to get out of there. Is it fair to your unborn child to be brought into this sort of situation? It is amazing the sort of things a baby can pick up from it's parents.
Start keeping a diary off all events, fights, dramas, etc so if things do end up in court you have something to use against her. Her mental health history could also work against her in a court. Though again go and get professional advice.
Do not rise to the arguments. Try to keep your cool, I know it is easier said then done, but the less you rise to her fights, the less she has to use against you
*

- Upvote
- Downvote
- Ignore

Please give a brief explanation for your decision.

New Page

18. Please rate or ignore the response comment (shown in blue).

/r/aww

Original poster:

My sister adopted this puppy with the most beautiful green eyes. This is him posing for me in a little dirt hole.

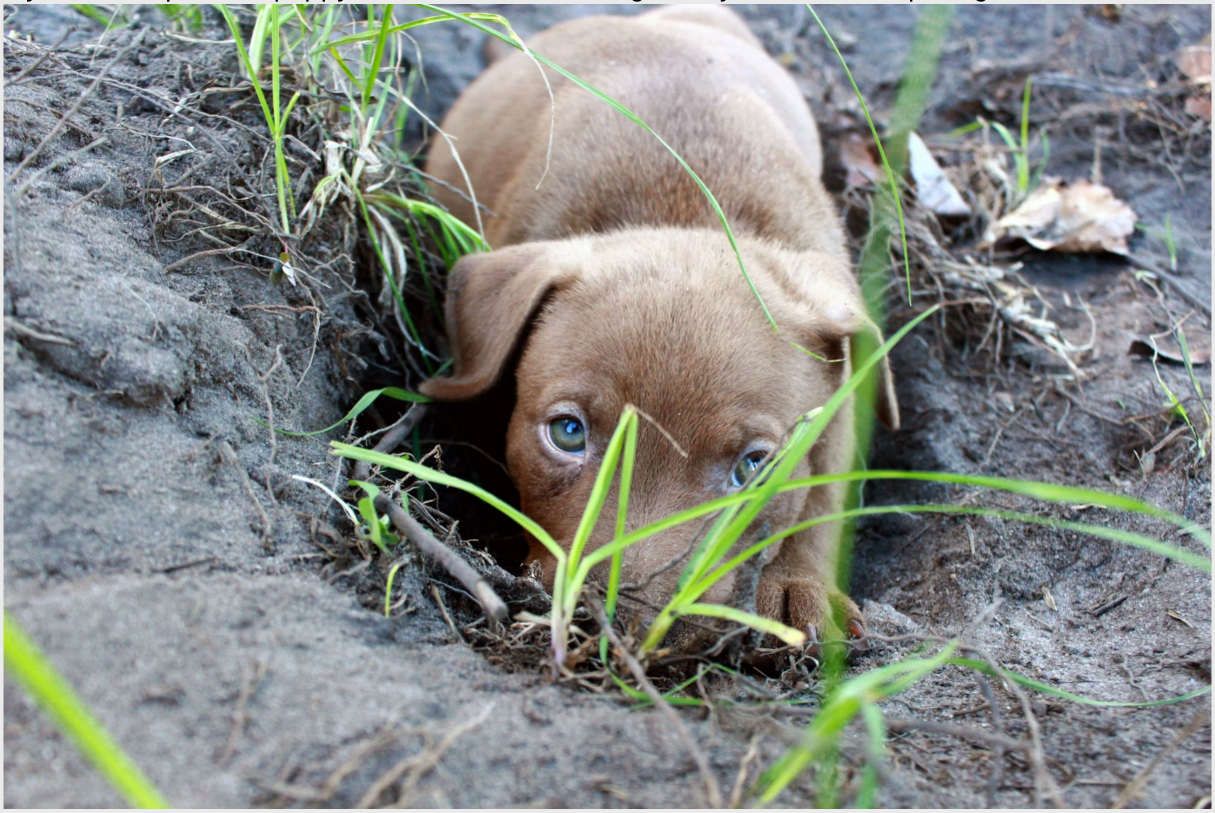

Response from OP:

He'll be growing up on a farm with:

- Butterball, his feisty brother
- Izzie, the crazy hyper one
- Onyx, the wise and loyal older one
- Brownie and Baby Cutie, the goats that act like puppies
- Rasta the rooster

and tons of chickens and ducks!

*

- Upvote
- Downvote
- Ignore

Please give a brief explanation for your decision.

New Page

19. Please rate or ignore the response comment (shown in blue).

/r/depression

Original poster:

I was feeling alone today and browsing a thread when I stumbled across this beautiful comic

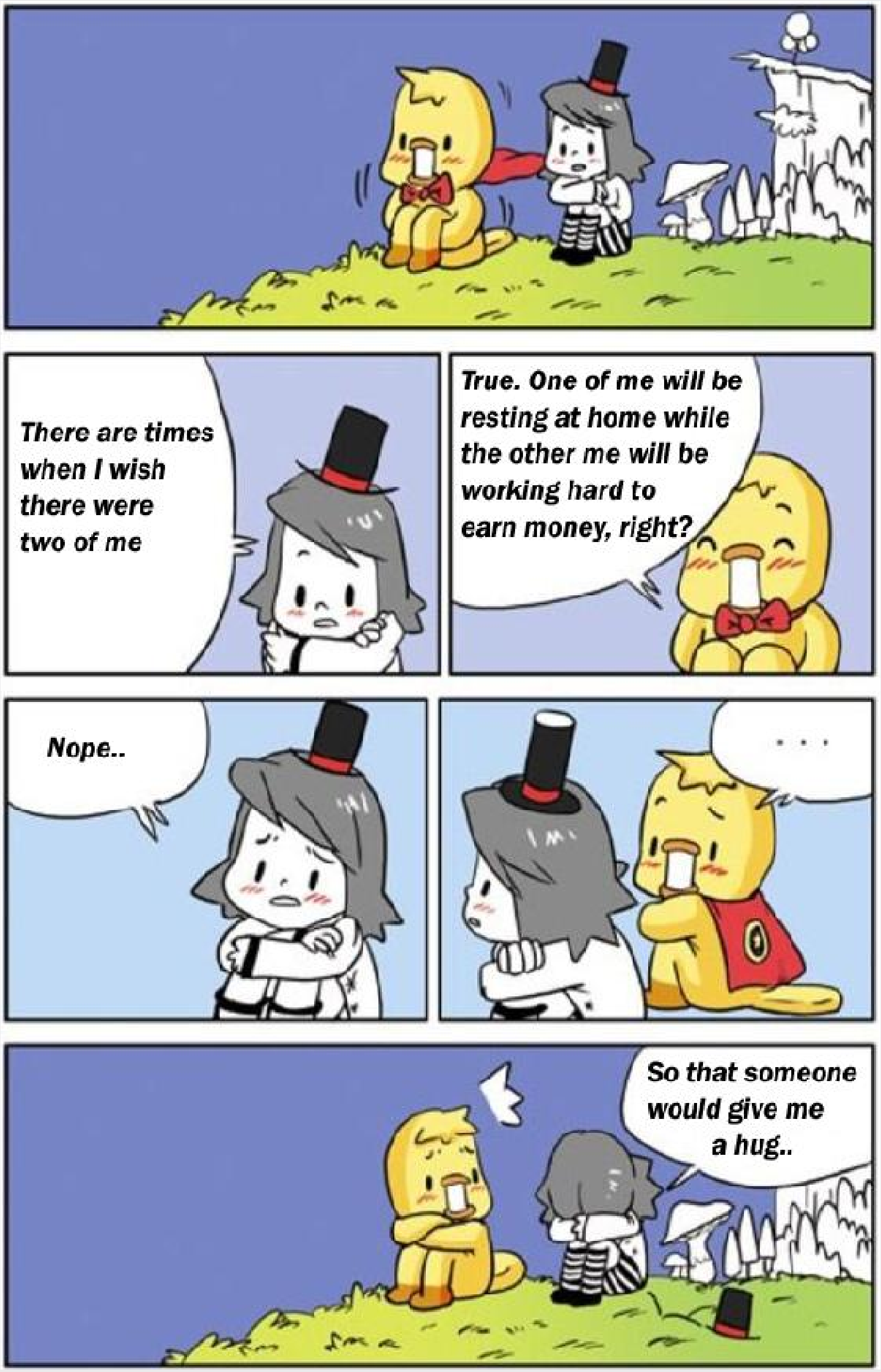

Responder:

I've felt like this lately :/.

*

- Upvote
- Downvote

○ Ignore

Please give a brief explanation for your decision.

[ ]

New Page

20. Please rate or ignore the response comment (shown in blue).

/r/AskReddit
Original poster:
Reddit what is your greatest achievement?
Responder:
I saved a kid from drowning once.
*

○ Upvote

○ Downvote

○ Ignore

Please give a brief explanation for your decision.

[ ]

New Page

21. Please rate or ignore the response comment (shown in blue).

/r/itookapicture
Original poster:
ITAP of the night sky while my town's power was out. Beautiful.

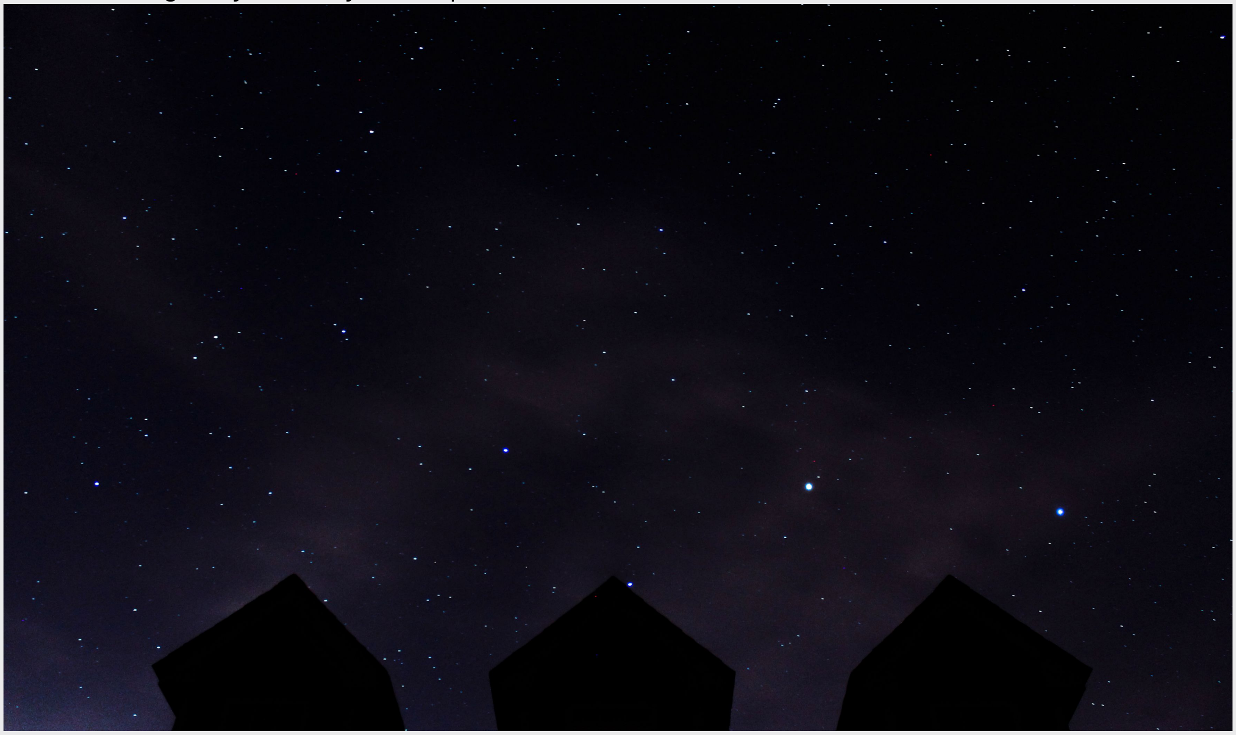

Responder:
It's almost sad that we can't see the stars in big cities.
*

- Upvote
- Downvote
- Ignore

Please give a brief explanation for your decision.

New Page

The following questions are designed to find out what motivates your voting behaviour on Reddit. Please answer as truthfully as possible.

22. How important are the following characteristics in helping you to decide to upvote something? *

	1 (not important)	2	3	4	5 (very important)
The content presents a generally-accepted opinion					
The content contains an interesting or unique perspective					
The content contains an opinion you agree with					
The content refers to something you have experienced (e.g. an event or a memory)					
The content features humour					
The content seems worthy of sympathy or support					
The content shows consideration for the good of other people and society					
The content is relevant to the post or subreddit					
The content sounds intelligent					
The content follows the rules of the subreddit					
The content contains something you wish other people to see					
The content is posted by a user you like					
The content already has a number of upvotes					
The content has a number of downvotes					

New Page

23. How important are the following characteristics in helping you to decide to downvote something? *

	1 (not important)	2	3	4	5 (very important)
The content contains reposted or unoriginal content	○	○	○	○	○
The content contains an opinion you disagree with	○	○	○	○	○
The content refers to something you have not experienced	○	○	○	○	○
The content brings back bad personal memories	○	○	○	○	○
The content features bad humour	○	○	○	○	○
The content is rude or aggressive	○	○	○	○	○
The content sounds immoral or damaging to society	○	○	○	○	○
The content is not relevant to the post or subreddit	○	○	○	○	○
The content sounds unintelligent	○	○	○	○	○
The content does not follow the rules of the subreddit	○	○	○	○	○
The content contains something you do not wish other people to see	○	○	○	○	○
The content is posted by a user you dislike	○	○	○	○	○
The content already has a number of downvotes	○	○	○	○	○
The content has a number of upvotes	○	○	○	○	○
The content seems to expect upvotes / is Karma-whoring	○	○	○	○	○

New Page

24. Are there any other factors which influence your decision to upvote or downvote something?

New Page

25. To what extent are your rating decisions influenced by the following factors? *

	1 (not at all)	2	3	4	5 (a lot)
An emotional reaction to the content	○	○	○	○	○
An objective evaluation of the contents' quality	○	○	○	○	○
The online reputation of the poster	○	○	○	○	○
The length of time the poster has been a Reddit user	○	○	○	○	○

26. Do you take more notice of highly upvoted content? *

○ Yes

○ No

27. Do you take more notice of content accompanied by Gold badges? *

○ Yes

○ No

Thank You!

Thank you very much for completing the survey. Your response is very important to us.

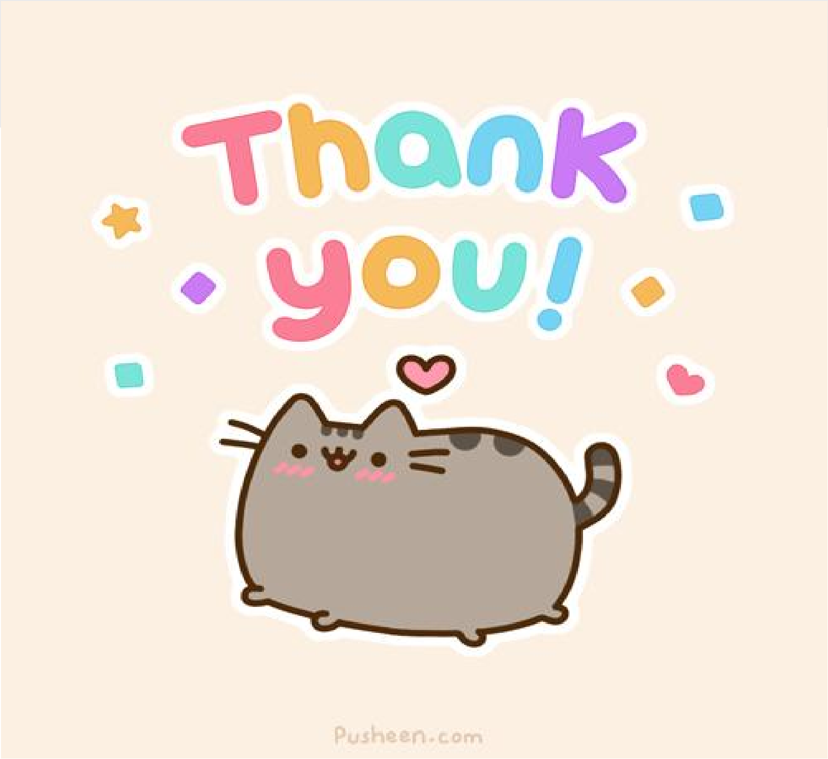
Thank you!
Pusheen.com
