# Supplementary material for: Do Online Voting Patterns Reflect Evolved Features of Human Cognition? An Exploratory Empirical Investigation
Source: PLoS One. 2015 Jun 11;10(6):e0129703. doi: 10.1371/journal.pone.0129703 (PMC4466230; doi:10.1371/journal.pone.0129703)
Supplement: S1 Table — (DOC) [file pone.0129703.s003.doc]

**Table S1.** Mean importance scores and standard deviations for voting influences.

|  | **Variable Mean** | **SD** | **Factor Mean** | **SD** |
| --- | --- | --- | --- | --- |
| *Wish Others To See (U)* | 4.27 | 1.00 |  |  |
| *Intelligence / Uniqueness* |  |  | 3.85 | 0.79 |
| Sounds intelligent (U) | 3.85 | 1.02 |  |  |
| Sounds unintelligent (D) | 3.48 | 1.25 |  |  |
| Interesting or unique (U) | 4.21 | 0.84 |  |  |
| *Reddit Norms* |  |  | 3.71 | 1.11 |
| Follows subreddit rules (U) | 3.44 | 1.33 |  |  |
| Doesn't follow subreddit rules (D) | 3.41 | 1.41 |  |  |
| Irrelevant to post or subreddit (D) | 3.78 | 1.31 |  |  |
| Relevant to post or subreddit (U) | 4.19 | 1.07 |  |  |
| *Prosociality* |  |  | 3.51 | 0.90 |
| Immoral or socially damaging (D) | 3.52 | 1.35 |  |  |
| Rude or aggressive (D) | 4.27 | 1.08 |  |  |
| Considerate of others (U) | 3.65 | 1.18 |  |  |
| Bad humour (D) | 3.15 | 1.36 |  |  |
| Shouldn’t be seen by others (D) | 2.94 | 1.50 |  |  |
| *Unoriginality* |  |  | 3.21 | 1.21 |
| Expects upvotes (D) | 3.21 | 1.43 |  |  |
| Reposted or unoriginal (D) | 3.21 | 1.40 |  |  |
| *Empathy / Humour* |  |  | 2.92 | 0.79 |
| Shared experiences (U) | 3.18 | 1.22 |  |  |
| Humour (U) | 3.55 | 1.08 |  |  |
| Elicits sympathy or support (U) | 3.01 | 1.28 |  |  |
| Agreement (U) | 3.27 | 1.13 |  |  |
| Generally accepted opinion (U) | 1.63 | 0.88 |  |  |
| *Disagreement Of Opinion (D)* | 2.69 | 1.23 |  |  |
| *Social Influence* |  |  | 1.66 | 0.76 |
| Number of downvotes (U) | 1.76 | 1.00 |  |  |
| Number of upvotes (U) | 1.62 | 0.95 |  |  |
| Number of upvotes (D) | 1.71 | 1.08 |  |  |
| Number of downvotes (D) | 1.55 | 0.91 |  |  |
| *Attitude Towards User* |  |  | 1.60 | 0.83 |
| Posted by a disliked user (D) | 1.59 | 1.02 |  |  |
| Posted by a liked user (U) | 1.62 | 0.99 |  |  |
| *Unshared Experience / Bad Memories* |  |  | 1.49 | 0.74 |
| Unshared experience (D) | 1.46 | 0.78 |  |  |
| Brings back bad memories(D) | 1.53 | 0.93 |  |  |
